# Isolated Hepatic Perfusion With Melphalan for Patients With Isolated Uveal Melanoma Liver Metastases: A Multicenter, Randomized, Open-Label, Phase III Trial (the SCANDIUM Trial)

**DOI:** 10.1200/JCO.22.01705

**Published:** 2023-03-20

**Authors:** Roger Olofsson Bagge, Axel Nelson, Amir Shafazand, Charlotta All-Eriksson, Christian Cahlin, Nils Elander, Hildur Helgadottir, Jens Folke Kiilgaard, Sara Kinhult, Ingrid Ljuslinder, Jan Mattsson, Magnus Rizell, Malin Sternby Eilard, Gustav J. Ullenhag, Jonas A. Nilsson, Lars Ny, Per Lindnér

**Affiliations:** ^1^ Sahlgrenska Center for Cancer Research, Department of Surgery, Institute of Clinical Sciences, Sahlgrenska Academy, University of Gothenburg, Gothenburg, Sweden; ^2^ Department of Surgery, Sahlgrenska University Hospital, Gothenburg, Sweden; ^3^ Wallenberg Centre for Molecular and Translational Medicine, University of Gothenburg, Gothenburg, Sweden; ^4^ Department of Oncology, Institute of Clinical Sciences, Sahlgrenska Academy at University of Gothenburg, Sahlgrenska University Hospital, Gothenburg, Sweden; ^5^ Department of Radiology, Alingsås Hospital, Alingsås, Sweden; ^6^ Department of Ophthalmology, Mölndal Hospital, Sahlgrenska University Hospital, Gothenburg, Sweden; ^7^ Transplant Institute, Institute of Clinical Sciences, Sahlgrenska Academy at University of Gothenburg, Sahlgrenska University Hospital, Gothenburg, Sweden; ^8^ Department of Oncology and Department of Clinical and Biomedical Sciences, Linköping University, Linköping, Sweden; ^9^ Department of Oncology, Karolinska University Hospital, Stockholm, Sweden; ^10^ Department of Ophthalmology, Rigshospitalet, Copenhagen University Hospital Copenhagen, Copenhagen, Denmark; ^11^ Department of Oncology, Skåne University Hospital, Lund, Sweden; ^12^ Department of Radiation Sciences, Oncology, Umeå University Hospital, Umeå, Sweden; ^13^ Department of Immunology, Genetics and Pathology (IGP), Science for Life Laboratories, Uppsala University, Uppsala, Sweden; ^14^ Department of Oncology, Uppsala University Hospital, Uppsala, Sweden; ^15^ Harry Perkins Institute of Medical Research, University of Western Australia, Perth, WA, Australia

## Abstract

About half of patients with metastatic uveal melanoma present with isolated liver metastasis, in whom the median survival is 6-12 months. The few systemic treatment options available only moderately prolong survival. Isolated hepatic perfusion (IHP) with melphalan is a regional treatment option, but prospective efficacy and safety data are lacking.

**METHODS:**

In this multicenter, randomized, open-label, phase III trial, patients with previously untreated isolated liver metastases from uveal melanoma were randomly assigned to receive a one-time treatment with IHP with melphalan or best alternative care (control group). The primary end point was overall survival at 24 months. Here, we report the secondary outcomes of response according to RECIST 1.1 criteria, progression-free survival (PFS), hepatic PFS (hPFS), and safety.

**RESULTS:**

Ninety-three patients were randomly assigned, and 87 patients were assigned to either IHP (n = 43) or a control group receiving the investigator's choice of treatment (n = 44). In the control group, 49% received chemotherapy, 39% immune checkpoint inhibitors, and 9% locoregional treatment other than IHP. In an intention-to-treat analysis, the overall response rates (ORRs) were 40% versus 4.5% in the IHP and control groups, respectively (*P* < .0001). The median PFS was 7.4 months versus 3.3 months (*P* < .0001), with a hazard ratio of 0.21 (95% CI, 0.12 to 0.36), and the median hPFS was 9.1 months versus 3.3 months (*P* < .0001), both favoring the IHP arm. There were 11 treatment-related serious adverse events in the IHP group compared with seven in the control group. There was one treatment-related death in the IHP group.

**CONCLUSION:**

IHP treatment resulted in superior ORR, hPFS, and PFS compared with best alternative care in previously untreated patients with isolated liver metastases from primary uveal melanoma.

## INTRODUCTION

Uveal melanoma is a rare disease accounting for approximately 3% of all melanomas. Even when the primary tumor is successfully eradicated from the eye with surgery or radiotherapy, approximately 50% of patients will develop metastases.^1^ Metastases are strongly hepatotropic, with isolated liver metastases seen in over half of patients with metastatic disease. Among these patients, the median survival is approximately 10-12 months and only a few patients survive more than 5 years.^1,2^ These disappointing outcomes reflect generally poor responses to systemic chemotherapy, which shows minimal efficacy and delivers no detectable survival benefit.

**CONTEXT**

**Key Objective**
Does isolated hepatic perfusion improve outcomes in previously untreated patients with liver metastases from uveal melanoma?
**Knowledge Generated**
In this randomized controlled multicenter phase III trial, 93 patients were randomly assigned to either isolated hepatic perfusion or a control group. In an intention-to-treat analysis, both the overall response rate and progression-free survival were significantly and substantially improved, favoring isolated hepatic perfusion over the control arm.
**Relevance *(R.G. Maki)***
While overall survival data are not presented, the benefit and toxicity associated with isolated hepatic perfusion for metastatic uveal melanoma are quantified, which will be useful both now and for future studies of therapy for this rare form of melanoma.*
*Relevance section written by *JCO* Associate Editor Robert G. Maki, MD, PhD, FACP, FASCO.

In contrast to cutaneous melanoma, immune checkpoint inhibition (ICI) has been of only limited benefit in patients with uveal melanoma, with the combination of ipilimumab and nivolumab showing an overall response rate (ORR) of 10%-18% and an uncertain impact on survival.^3-5^ The combination of pembrolizumab and epigenetic therapy with a histone deacetylase inhibitor resulted in durable responses in a subset of patients.^6^ More encouragingly, a recent randomized phase III trial of tebentafusp, a bispecific fusion protein linking melanoma cells with T cells in HLA-A*02:01 serotype patients with metastatic uveal melanoma, resulted in a significant improvement in overall survival (OS; 22 *v* 16 months).^3^

For patients with isolated liver metastases, several locoregional liver treatment strategies have been investigated. A retrospective analysis of highly selected patients undergoing surgery and achieving an R0 resection reported a median survival of 27 months.^4^ Some patients with uveal melanoma have also responded to transarterial chemoembolization^5^ and selective internal radiation therapy in small cohort studies.^7^ However, most patients treated locoregionally ultimately progress, probably because of a silent miliary pattern of disease initially not detected radiologically.

Isolated hepatic perfusion (IHP) with melphalan is a regional treatment where the liver is completely isolated from the systemic circulation to allow hepatic perfusion with high concentrations of chemotherapy, with minimal systemic exposure.^8^ IHP has been evaluated in several studies, mainly for liver metastases derived from colorectal cancer, melanoma, neuroendocrine tumors, and primary hepatic malignancies.^9-11^ A retrospective study comparing patients with uveal melanoma metastases treated with IHP with the longest surviving patients in Sweden during the same time period showed a 14-month increase in survival (26 *v* 12 months) in the IHP group.^12^

In this randomized phase III trial, we compared IHP with the investigator's choice of therapy as first-line treatment in patients with isolated uveal melanoma liver metastases. In this preplanned analysis, we report the end points of ORR, hepatic progression-free survival (hPFS), progression-free survival (PFS), and safety in terms of serious adverse events (SAEs).

## METHODS

### Patients

Patients with histologically or cytologically confirmed liver metastases from uveal melanoma were eligible if they had measurable disease according to RECIST version 1.1 criteria, had received no previous therapy for melanoma metastases (ie, first-line therapy), and had no evidence of extrahepatic disease by positron emission tomography-computed tomography (CT). Patients were excluded if tumor occupied 50% or more of the liver as assessed by CT or magnetic resonance imaging (MRI); if there was significant heart, lung, or renal dysfunction; or if the patient had a BMI above 35.

### Study Design and Treatment

This was a prospective, multicenter, open-label, phase III trial randomly assigning patients in a 1:1 ratio to IHP or control (investigator's choice of treatment). Random assignment was performed using permutated blocks with variable size stratified for study site. Inclusion in the trial was recommended as the first-line treatment option by the national Swedish guidelines and as such can be seen as a population-based study.

IHP was performed at Sahlgrenska University Hospital, Gothenburg, Sweden. The procedure has previously been described in detail.^13^ In summary, a shunt was inserted into the iliac and jugular veins and connected to an external centrifugal pump to allow for shunting of blood from the lower extremity during the procedure after clamping the vena cava. The vena cava was isolated infrahepatically above the renal veins and suprahepatically between the liver and the diaphragm. A catheter was placed in the retrohepatic portion of the vena cava for perfusion outflow, and the hepatic artery proper was cannulated for perfusion inflow. The portal vein was clamped, and the catheters were connected to a perfusion system. Perfusion was performed with a target flow rate of 500-1,200 mL/min with a target liver temperature of 40°C. When steady-state conditions in the perfusion circuit were established, melphalan at a dose of 1 mg/kg body weight was added to the perfusion system, divided into two doses given 30 minutes apart. Perfusion was continued for 60 minutes, after which the perfusion was discontinued and the liver irrigated (Ringer Acetat, Baxter, Sweden). The shunts and the perfusion circuit were disconnected, and the catheters were removed.

Patients randomly assigned to the IHP group were not allowed to receive any other antitumoral treatment during follow-up until progression. Patients randomly assigned to the control group received the investigator's choice of treatment according to the discretion of the treating physician at each study site. All available treatments including surgery and other experimental treatments were accepted; however, no crossover to IHP was allowed.

### Study Assessments

Radiologic response was assessed after 3, 6, 12, 18, and 24 months by either CT or MRI of the liver using the same modality as at the baseline examination, with additional CT imaging of the thorax. Response was assessed on the basis of blinded independent central review according to RECIST version 1.1.^14^ Safety was assessed by collecting data for SAEs. Laboratory values and vital signs were assessed regularly and graded according to the National Cancer Institute Common Terminology Criteria for Adverse Events version 4.0.

### End Points

The primary end point was OS at 24 months, but in this first preplanned analysis, the secondary end points of ORR, hPFS, and SAEs and exploratory end points of PFS and duration of response (DOR) were reported.

ORR was defined as the proportion of patients who had a partial or complete response (CR) to therapy. PFS was defined as the time from random assignment to documented progression at any site or death from any cause, and hPFS from random assignment to progression in the liver or death from any cause, both according to RECIST 1.1 criteria. ORR was defined as the percentage of patients with a best overall response (BOR) of CR or partial response (PR) according to RECIST. DOR was defined as the time from the first documented response to radiologic progression according to RECIST 1.1 criteria. Efficacy was assessed in the intention-to-treat population, with all patients included in the treatment group to which they were randomly assigned.

SAEs were assessed in the per-protocol (PP) population, which was defined as all patients who underwent random assignment and were grouped according to the treatment that they received. The observation time in this analysis was defined from random assignment to the time point of starting second-line therapy or death from any cause.

### Statistical Analysis

The sample size was based on assumptions of an estimated treatment effect with 50% survival in the study group and 20% survival in the control group after a 24-month follow-up. To reach a power of 80% with an α value of 0.05 and a treatment group ratio of 1:1 and using two-sided Fisher's exact test, a sample size of 90 patients (45 patients per study arm) was required. After an interim report from the data safety monitoring board, it was recommended to include an additional three patients to compensate for dropout, that is, a total of 93 patients.

Time-to-event analysis was performed on all patients in the intention-to-treat (ITT) and PP populations using the Kaplan-Meier methodology and is reported using medians together with 95% CIs and estimated survival rates at 6, 12, and 24 months with 95% CI. Fisher's exact test was used to compare ORR.

### Study Oversight

The original protocol and all amendments were approved by the Swedish Medical Product Agency (EudraCT No. 2013-000564-29) and the Regional Ethical Review Board at the University of Gothenburg (Dnr 144-13). The study was registered at ClinicalTrials.gov (identifier: NCT01785316). The study was conducted in accordance with the protocol, Good Clinical Practice guidelines, and the provisions of the Declaration of Helsinki. All patients provided written informed consent before inclusion in the trial.

## RESULTS

### Patients

**FIG 1.**
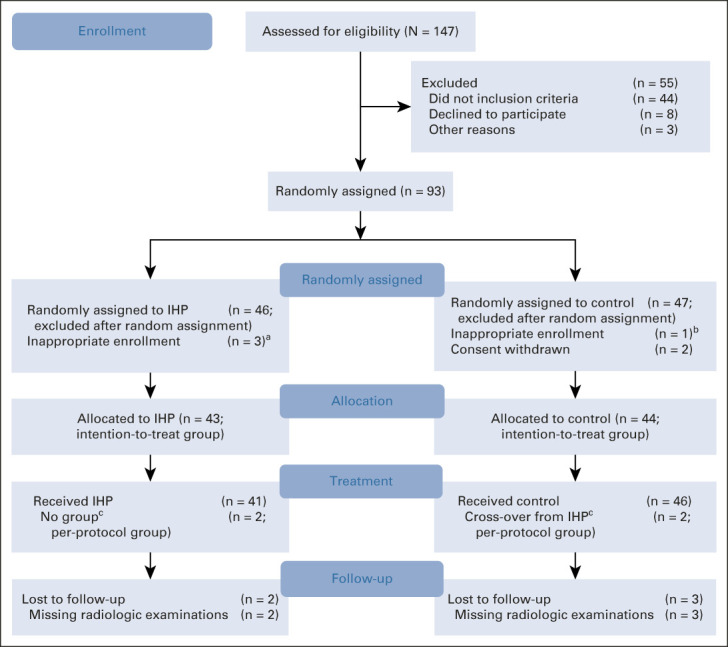
CONSORT diagram. ^a^Three patients were inappropriately enrolled in the IHP arm, two patients because of > 50% of the liver being occupied with metastasis and one patient because of the presence of systemic metastases. ^b^One patient was inappropriately enrolled in the control arm because liver metastases were not verified by biopsy. ^c^Two patients did not receive IHP since their tumor burden was estimated to be >50% after laparotomy and perioperative evaluation, so these two patients then crossed over to the control group. IHP, isolated hepatic perfusion.

From July 2013 to March 2021, 147 patients were screened and 93 patients were enrolled at six sites and randomly assigned to the IHP group (46 patients) or the control group (47 patients). Six patients were excluded after random assignment but before treatment initiation, four because of not fulfilling the inclusion criteria (two patients because of metastasis occupying >50% of the liver, one patient because of the presence of systemic metastases, and one patient where the metastasis was not verified by biopsy), and two patients withdrew their consent (Fig 1). The median age was 65 years (range, 27-80) in the IHP group and 68 years (range, 40-85) in the control group, and the demographics and baseline disease characteristics of the patients are given in Table 1. The median duration of follow-up at the time of data cutoff for this interim analysis (March 10, 2022) was 18.6 months (range, 1.4-24.0 months).

**TABLE 1.**
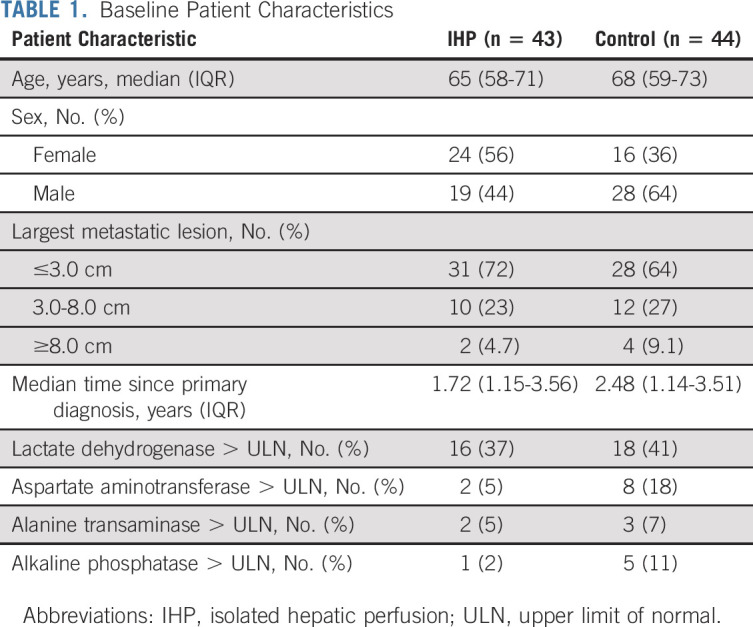
Baseline Patient Characteristics

Among patients in the IHP group, 41 (89%) were treated PP and two (4.5%) did not receive IHP because of the perioperative observation that >50% of the liver was occupied with metastases, so these patients immediately crossed over to the control arm. In the IHP group, the median operating time was 5.0 hours (range, 1.0-8.0 hours) and the median hospital stay was 7 days (range, 4-21 days). The median perioperative liver temperature was 40°C (range, 39°C-40°C), the median perfusion flow rate was 600 mL/min (range, 50-1,020 mL/min), and the median blood loss was 600 mL (range, 0-2,400 mL). Among the patients in the control group, the first-line treatment was chemotherapy in 21 (48%) patients, ICIs in 17 (39%) patients, and localized treatment interventions in five (11%) patients. One (3%) patient did not receive any antitumor treatment because of clinical deterioration (Table 2).

**TABLE 2.**
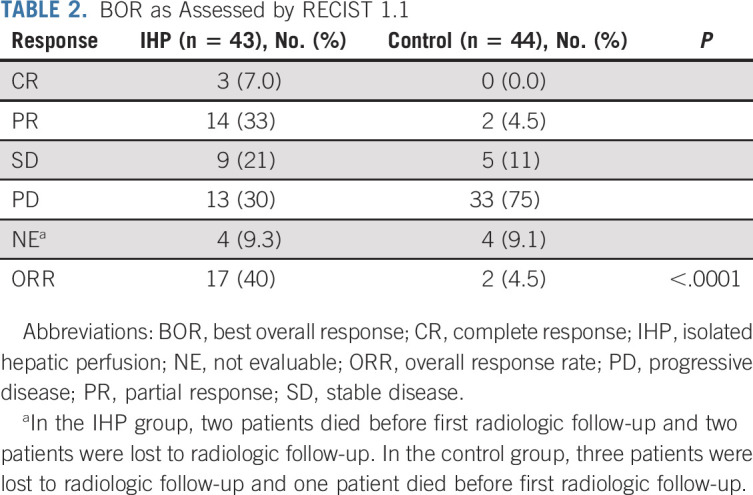
BOR as Assessed by RECIST 1.1

### Efficacy

**FIG 2.**
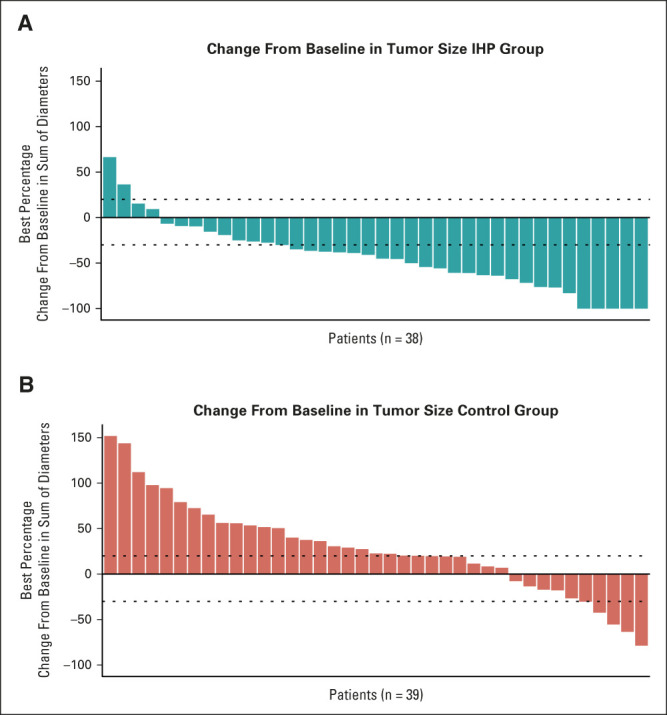
Waterfall plots for the isolated hepatic perfusion and control group. Shown are the best percentage changes from baseline in the sum of the largest diameters of measurable tumors in patients for whom data from both baseline and postbaseline assessments of target lesions by independent central review were available: (A) 38 of 43 patients who received IHP and (B) 39 of 44 of patients who received control treatment. The upper dashed horizontal line indicates a 20% increase in tumor size in the patients who had disease progression, and the lower dashed line indicates a 30% decrease in tumor size (PR). IHP, isolated hepatic perfusion; PR, partial response.

Treatment with IHP was assessed by central radiologic review, and there was an objective response in 17 patients (40% ORR) receiving IHP compared with two patients (4.5%) receiving control treatment (*P* < .0001, Table 3). Three patients (7%) in the IHP group experienced a CR compared with no patient in the control group. Progressive disease (PD) was the BOR in 13 patients (30%) in the IHP group and 33 patients (75%) in the control group. The percentage of patients achieving disease control (defined as CR, PR, or stable disease [SD] for at least 3 months) was 58% in the IHP group and 15% in the control group (Fig 2).

**TABLE 3.**
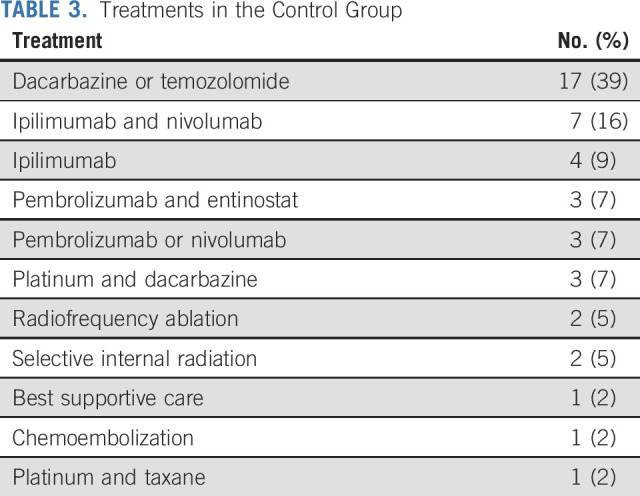
Treatments in the Control Group

**FIG 3.**
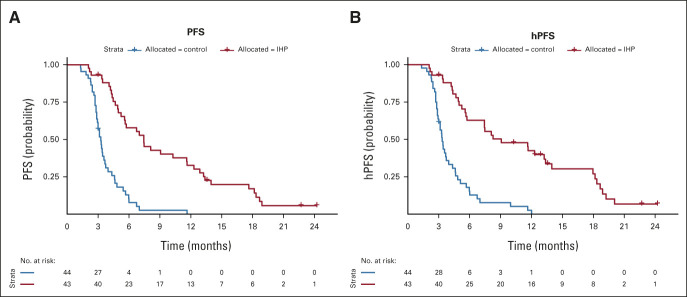
Kaplan-Meier estimates of PFS and hPFS comparing isolated hepatic perfusion and control. Kaplan-Meier estimates of (A) PFS and (B) hPFS as assessed by blinded central review in the ITT population. The tick marks indicate censored data. hPFS, hepatic progression-free survival; IHP, isolated hepatic perfusion; ITT, intention-to-treat; PFS, progression-free survival.

The estimated 6-month PFS rates were 58% for the IHP group compared with 8% in the control group. The median PFS was 7.4 months (95% CI, 5.2 to 11.6) in the IHP group compared with 3.3 months (95% CI, 2.9 to 3.7; *P* < .0001) in the control group, with a hazard ratio (HR) of 0.21 (95% CI, 0.12 to 0.36; Fig 3A). The median PFS in the control group was 3.0 months for patients receiving chemotherapy and 3.3 months for patients receiving ICIs.

The estimated 6-month hPFS rates were 63% for the IHP group compared with 13% in the control group. The median hPFS was 9.1 months (95% CI, 5.6 to 13.4) in the IHP group compared with 3.3 months in the control group (95% CI, 2.9 to 4.0, *P* < .0001), with a HR of 0.21 (95% CI, 0.12-0.36; Fig 3B). The median hPFS in the control group was 3.0 months (95% CI, 2.8 to 4.3) for patients receiving chemotherapy and 3.3 months (95% CI, 2.8 to 5.1) for patients receiving ICIs.

The median DOR was 13.7 months (95% CI, 11.6 to 18.3) in the IHP group (n = 17) compared with 8.8 months (95% CI, 6.0 to not evaluable) in the control group (n = 2). In patients randomly assigned to IHP with a BOR of CR, PR, or SD who later progressed (n = 23), the site for progression was hepatic in 12 (52%), extrahepatic in six (26%), and simultaneous hepatic and extrahepatic progression in five (22%) patients. Among IHP patients who were primarily resistant to IHP (ie, BOR of PD, n = 13), 12 (92%) had primary progression in the liver, whereas only one patient (8%) had extrahepatic progression.

### Safety

SAEs occurring during first-line therapy (from random assignment to the time of starting second line therapy) were reported in eight (19.5%) patients in the IHP group and three (6.5%) in the control group (summarized in Table 4). There was one treatment-related death in the IHP group, where the patient had a liver artery dissection that was detected seven days after IHP and who died 16 days after IHP because of multiorgan failure secondary to liver artery thrombosis causing liver necrosis and aspiration pneumonia, confirmed by autopsy. All SAEs reported in the IHP group occurred within 3 months of IHP.

**TABLE 4.**
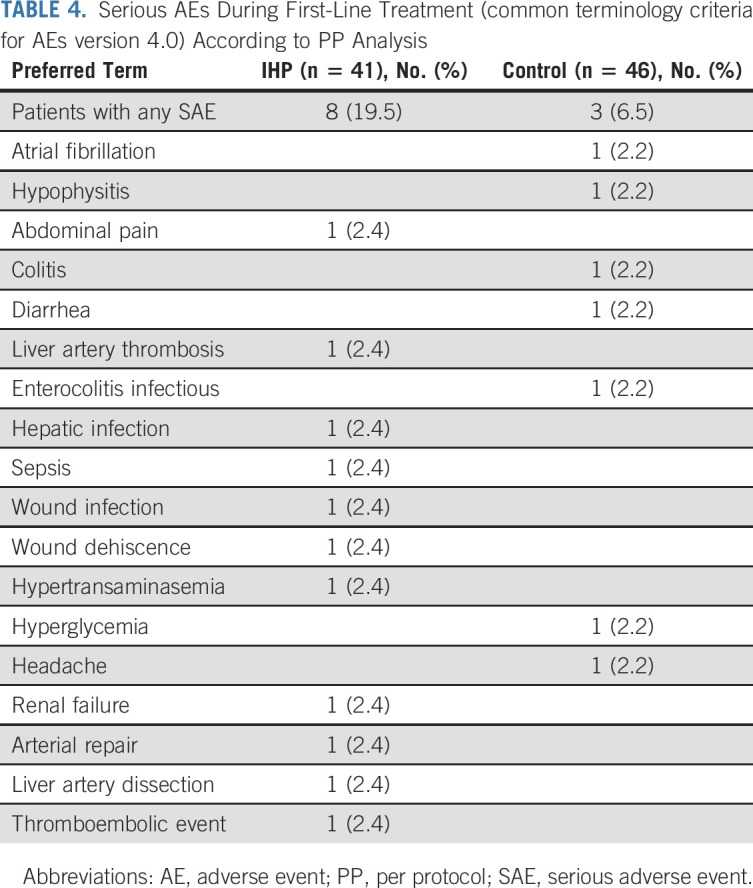
Serious AEs During First-Line Treatment (common terminology criteria for AEs version 4.0) According to PP Analysis

## DISCUSSION

In this, to our knowledge, first analysis of a randomized controlled phase III trial of previously untreated patients with isolated uveal melanoma liver metastases, a single treatment with IHP with melphalan was well tolerated with a manageable toxicity profile and resulted in significantly higher response rates and improved hPFS and PFS than the investigator's choice of available treatments.

The outcomes in control group patients were similar to those reported in other recent studies, confirming that the control group is representative, and there was no cross-over allowing for IHP in the control arm. The ORR was 4.5%, and the median PFS was 3.3 months in the control arm, similar to previously reported results on patients with metastatic uveal melanoma treated with chemotherapy or ICIs with a CTLA-4 or a PD-1 inhibitor.^15^ Recently, several phase II trials have reported an ORR of 10%-18% in patients with metastatic uveal melanoma treated with ipilimumab and nivolumab.^3-5,16-18^ Seven patients in the control arm of the present trial received combination of ICI with ipilimumab and nivolumab, one of whom experienced a PR. The recent approval of tebentafusp for the treatment of HLA-A*02:01 subtype patients with uveal melanoma is a major advance. However, no control patients in the present trial received tebentafusp as the treatment was not available during the inclusion phase of the trial. It is a limitation that the control group of the present study was treated heterogeneously, and the study is underpowered for any direct comparison between the individual treatments.

Previous knowledge of outcomes after IHP is primarily based on smaller, retrospective single-institution series, where Alexander et al reported the pioneering studies on IHP for uveal melanoma metastases with an ORR of 62% including 10% with CR.^19,20^ Other series have shown similar results with the ORR ranging between 33% and 70% and CR rates between 0% and 7%.^21-23^ In the early 1990s, three independent groups developed the percutaneous hepatic perfusion (PHP) technique, which combined conventional hepatic artery infusion with a dual-balloon vena cava catheter collecting the outflow from the liver. The venous outflow was then connected to an extracorporeal venous bypass circuit including a carbon filter to recover any of the drug that was not absorbed by the liver.^24-26^ A phase III study randomly assigned 93 patients to either PHP or best alternative care, which demonstrated a significantly better hPFS (245 days *v* 49 days) and ORR (34% *v* 2%) in the PHP group. However, it was difficult to draw conclusions concerning OS in this study as a high proportion of patients crossed over from best alternative care to PHP and the study also included patients with both uveal and cutaneous melanoma not reported separately.^27,28^

A recent phase III study, the FOCUS trial, compared PHP with the investigator's choice of transarterial chemoembolization, ipilimumab, pembrolizumab, or dacarbazine in patients with metastatic uveal melanoma with hepatic-dominant disease.^29^ The trial started as a randomized trial where 43 patients were randomly assigned to PHP (40 patients received treatment) and 42 to the control arm, but because of enrollment concerns, the control arm was stopped and another set of 59 patients was assigned to the PHP arm (51 patients received treatment). The trial showed a very similar response, PFS, and DOR as the current trial, supporting the fact that liver-directed therapies do have substantial effects for these patients. Further support is given by data from a recent meta-analysis, where there was no difference in hPFS (10.0 *v* 9.5 months) or OS (17.1 *v* 17.3 months) between IHP and PHP for patients with uveal melanoma liver metastases. However, there were a higher complication rate (39.1% *v* 23.8%) and a higher 30-day mortality (5.5% *v* 1.8%) for IHP compared with PHP.^30^

An interesting development is the combination of IHP or PHP with systemic immunotherapy. Data have shown a correlation between OS and a high infiltration of CD8^+^ T cells in metastases and an activated immune cell profile in peripheral blood in patients treated with IHP,^31^ and ongoing studies are currently investigating the combination of CTLA-4 and PD-1 inhibitors with either PHP (CHOPIN trial ClinicalTrials.gov identifier: NCT04283890) or IHP (SCANDIUM-II trial ClinicalTrials.gov identifier: NCT04463368).

The incidence of SAEs was higher in patients receiving IHP compared with patients in the control group. The adverse events in the IHP group were mainly expected complications because of major surgery. However, there was one treatment-related death in the IHP group. The patient was severely obese, a factor discussed thoroughly by the investigators as a potential contributing cause of death, and a new inclusion criterion of a BMI < 35 was added. The risks associated with obesity and IHP are probably related to obesity being a risk factor for surgery in general and not directly to IHP specifically. In comparison with PHP,^29^ the incidence of SAEs was lower in our cohort of patients treated with IHP, but the events in our patients were probably more severe.

In summary, the present randomized controlled trial shows that a one-time treatment with IHP results in significantly superior antitumor responses and PFS compared with treatment with chemotherapy or ICIs in treatment-naïve patients with isolated liver metastases of uveal melanoma. The first analysis of OS, the primary end point of the study, is planned for 2023.
